# Reiterative Synthesis by the Ribosome and Recognition of the N-Terminal Formyl Group by Biosynthetic Machinery Contribute to Evolutionary Conservation of the Length of Antibiotic Microcin C Peptide Precursor

**DOI:** 10.1128/mBio.00768-19

**Published:** 2019-04-30

**Authors:** Inna Zukher, Michael Pavlov, Darya Tsibulskaya, Alexey Kulikovsky, Tatyana Zyubko, Dmitry Bikmetov, Marina Serebryakova, Satish K. Nair, Måns Ehrenberg, Svetlana Dubiley, Konstantin Severinov

**Affiliations:** a Institute of Gene Biology, Russian Academy of Science, Moscow, Russia; b Uppsala Biomedicinska Centrum BMC, Uppsala, Sweden; c Center for Life Sciences, Skolkovo Institute of Science and Technology, Moscow, Russia; d A.N. Belozersky Institute of Physico-Chemical Biology, Lomonosov Moscow State University, Moscow, Russia; e University of Illinois, Urbana, Illinois, USA; f Waksman Institute for Microbiology, Rutgers, Piscataway, New Jersey, USA; g Institute of Molecular Genetics, Russian Academy of Sciences, Moscow, Russia; Massachusetts Institute of Technology; University of Illinois at Urbana-Champaign; The Ohio State University; University of Illinois at Urbana-Champaign; Muséum national d'Histoire naturelle

**Keywords:** antibiotic, microcin, ribosome, translation initiation

## Abstract

Microcin C (McC) is a peptide adenylate antibiotic produced by Escherichiacoli cells bearing a plasmid-borne *mcc* gene cluster. Most MccA precursors, encoded by validated *mcc* operons from diverse bacteria, are 7 amino acids long, but the significance of this precursor length conservation has remained unclear. Here, we created derivatives of E. coli *mcc* operons encoding longer precursors and studied their synthesis and bioactivities. We found that increasing the precursor length to 11 amino acids and beyond strongly decreased antibiotic production. We found this decrease to depend on several parameters. First, reiterative synthesis of the MccA peptide by the ribosome was decreased at longer *mccA* open reading frames, leading to less efficient competition with other messenger RNAs. Second, the presence of a formyl group at the N-terminal methionine of the heptameric peptide had a strong stimulatory effect on adenylation by the MccB enzyme. No such formyl group stimulation was observed for longer peptides. Finally, the presence of the N-terminal formyl on the heptapeptide adenylate stimulated bioactivity, most likely at the uptake stage. Together, these factors should contribute to optimal activity of McC-like compounds as 7-amino-acid peptide moieties and suggest convergent evolution of several steps of the antibiotic biosynthesis pathway and their adjustment to sensitive cell uptake machinery to create a potent drug.

## Abstract (precis)

Escherichia coli microcin C (McC) is a representative member of peptide-nucleotide antibiotics produced by diverse microorganisms. The vast majority of biosynthetic gene clusters responsible for McC-like compound production encode 7-amino-acid-long precursor peptides, which are C-terminally modified by dedicated biosynthetic enzymes with a nucleotide moiety to produce a bioactive compound. In contrast, the sequences of McC-like compound precursor peptides are not conserved. Here, we studied the consequences of E. coli McC precursor peptide length increase on antibiotic production and activity. We show that increasing the precursor peptide length strongly decreases McC production by affecting multiple biosynthetic steps, suggesting that the McC biosynthesis system has evolved under significant functional constraints to maintain the precursor peptide length.

## INTRODUCTION

Microcin C (McC) is a ribosomally synthesized and posttranslationally modified peptide (RiPP) produced by Escherichia coli cells bearing a plasmid-borne *mcc* gene cluster. McC is a heptapeptide adenylate that inhibits the growth of sensitive cells by a Trojan-horse mechanism. It is transported inside E. coli or other closely related bacterial cells by the YejABEF transporter, which specifically recognizes the peptide moiety of the prodrug (1). Inside the cell, the peptide is degraded by aminopeptidases, leading to the release of a toxic, nonhydrolyzable aspartamide adenylate (2). This compound structurally mimics aspartyl adenylate, an intermediate of a reaction catalyzed by aspartyl-tRNA synthetase (3). Inhibition of this essential enzyme by processed McC leads to an accumulation of uncharged tRNA^Asp^ and cessation of protein synthesis and growth (4).

**FIG 1:**
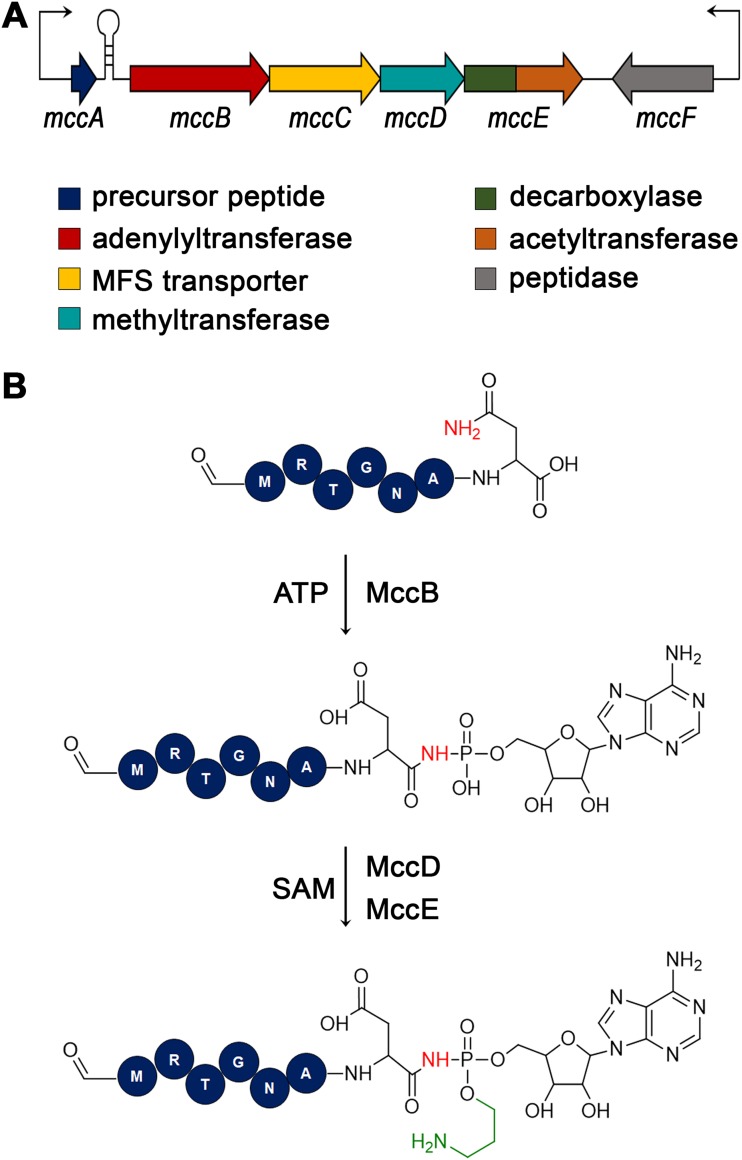
Escherichia coli *mcc* gene cluster and biosynthesis of microcin C. (A) The E. coli *mcc* biosynthetic gene cluster is schematically shown. Genes are shown by colored arrows and the functions of gene products are indicated below. Thin arrows indicate promoters from which transcription of *mcc* genes is initiated. A transcription terminator located between the *mccA* and *mccB* genes is shown as a hairpin. (B) The steps of the McC biosynthesis pathway and enzymes involved are presented. For the peptide part, the first 6 amino acids are shown as circles with their identity indicated in a single-letter amino acid code. The last amino acid is shown as a skeletal formula. The N-terminal methionine residue of mature McC is formylated.

The E. coli *mcc* gene cluster contains a five-gene operon, *mccABCDE*, and a nearby independently expressed *mccF* gene (Fig. 1). The 7-amino-acid-long McC precursor (MccA; MRTGNAN), the product of the *mccA* gene, is adenylated by the MccB enzyme in an ATP-dependent process (5). The adenosine moiety is attached to the C terminus of the peptide via a nonhydrolyzable N-P bond. The peptide adenylate produced by MccB is additionally decorated with an aminopropyl group attached to the phosphoramidate by the joint activity of MccD and the N-terminal domain of a bifunctional MccE enzyme (6). The C-terminal domains of MccE and MccF contribute to the autoimmunity of the peptide-adenylate-producing cells by detoxifying the processed McC that accumulates in their cytoplasm (7, 8). The *mccC* gene encodes the MccC pump protein required for the extrusion of mature McC as well as premature McC without the aminopropyl group.

Though the aminopropyl moiety increases the toxicity of McC by ca. 4- to 10-fold for some strains of E. coli, the peptide adenylate itself also inhibits bacterial growth (9). Thus, the minimal set of three genes in an *mcc* cluster, encoding the precursor peptide, the adenylating enzyme, and the export pump protein, should be sufficient for production of McC-like antibacterial compounds. Bioinformatics searches reveal that genes coding for MccB-like proteins can be found in various bacteria that are phylogenetically distant from E. coli (10).

Here, we report that extension of the gene encoding the E. coli MccA heptapeptide dramatically decreases peptide-nucleotide production. We show that the fitness gain of confining *mccA* to encode a heptapeptide depends on at least two parameters. First, *in vitro* ribosomal synthesis of the MccA heptapeptide proceeds via multiple rounds of *mccA* mRNA translation without the dissociation of the ribosome from the template, in line with earlier work on minigene expression (11). Ribosome recycling without mRNA dissociation is much less effective for open reading frames (ORFs) encoding longer MccA variants. We suggest that selective amplification of MccA heptapeptide synthesis compared to the synthesis of longer peptides results also in more efficient McC production inside the cell, where intracellular mRNAs compete with *mccA* mRNA for the ribosomes. Second, N-terminal formylation of the MccA heptapeptide promotes efficient adenylation by the MccB enzyme, and the formyl group on the heptapeptide adenylate specifically increases antibacterial activity. We speculate that these factors together contribute to the observed conservation of MccA homolog lengths in diverse bacteria.

## RESULTS

### Bioinformatics analysis.

We performed a bioinformatics search for genes encoding MccB-like proteins in the NCBI protein database. Manual searches revealed the existence of short genes encoding putative peptide precursors in the vicinity of most such *mccB* homologs. All precursors contained a C-terminal asparagine residue required for installation of the N-P bond by MccB, and their open reading frames were preceded by a strong Shine-Dalgarno sequence. Some such MccA-MccB pairs were previously validated by the reconstitution of enzymatic activity on chemically synthesized MccA peptide precursors by cognate MccB homologs *in vitro* (10).

**FIG 2:**
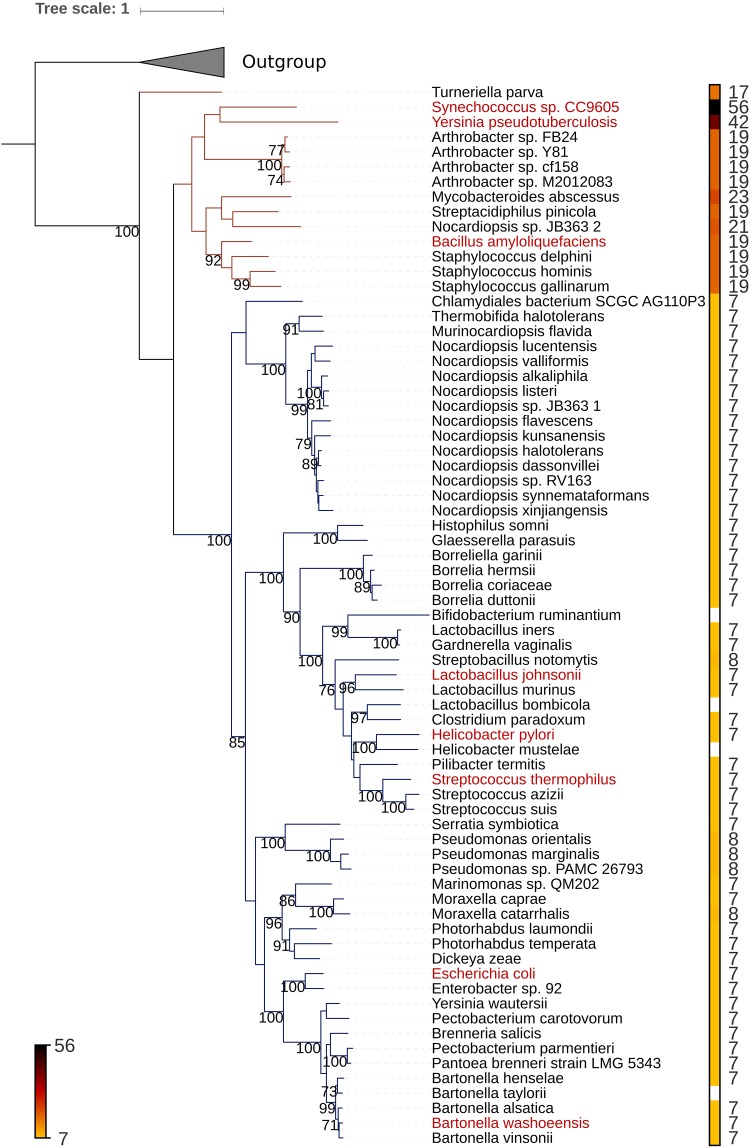
Maximum likelihood phylogenetic tree of MccB and MccB-like proteins. The unrooted tree was generated using RAxML (24) with 400 bootstrap replicates. The numbers at the nodes indicate the bootstrap values. Only bootstrap values greater than 70% are shown. Scale bar shows the number of inferred amino acid substitutions per site. Triangle marks the outgroup consisting of PaaA homologs not involved in the production of McC-like compounds. Each terminal node of the tree corresponds to the sequence representing a cluster of identical proteins and is labeled by the full systematic name of an organism (for the full data set see Table S1 in the supplemental material). The names of the organisms bearing the validated *mcc* clusters are shown in red. For other organisms, precursor peptides are putative and obtained by manual sequence analysis of *mcc*-like clusters. The lengths (in amino acids) of the verified or predicted microcin C precursor peptides are indicated both by labels and by the color strip. The color palette is given on the bottom left. White indicates that we were unable to predict the peptide. The blue and the red branches correspond to MccB subfamilies associated with 7-amino-acid and longer MccA precursors, respectively.

The phylogenetic tree of MccB homologs is presented in Fig. 2. The tree has a deeply rooted branch that separates MccB sequences into two major clades. An analysis of predicted and, in some cases, validated MccA precursors showed that they fall into two distinct groups. The vast majority of precursors are 7 (or more rarely 8) amino acids long and are associated with the major clade of MccB homologs (blue in Fig. 2). In the second much smaller group, the precursors are considerably longer, from 17 to 56 amino acids, and are associated with a different clade of MccB enzymes (red in Fig. 2).

10.1128/mBio.00768-19.1 TABLE S1 Accession numbers of MccB-like proteins used in phylogenetic analysis and corresponding MccA precursor peptides. Download Table S1, XLSX file, 0.1 MB. Copyright © 2019 Zukher et al. 2019 Zukher et al. This content is distributed under the terms of the Creative Commons Attribution 4.0 International license.

Apart from universally conserved N-terminal methionine and C-terminal asparagine, the sequences of the MccA peptide precursors associated with the major MccB clade differ greatly from each other (see Table S1 in the supplemental material). Their length, however, appears to be well conserved, suggesting high selection pressure for heptapeptide precursors among the biosynthetic clusters of McC-like compounds.

### Inefficient *in vivo* production of McC-like compounds with elongated peptide chains.

**FIG 3:**
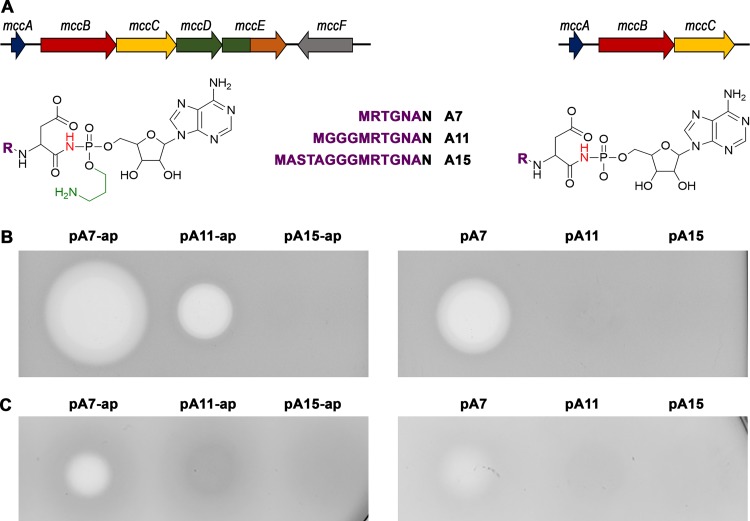
Bioactivity of secreted and intracellular McC and McC-like compounds. (A) Organization of gene clusters used in this study and structures of the expected compounds produced by complete and partial *mcc* gene clusters shown at the top. R, MRTGNA (pA7 and pA7-ap plasmids), MGGGMRTGNA (pA11 and pA11-ap plasmids), or MASTAGGGMRTGNA (pA15 and pA15-ap plasmids). (B) Growth-inhibiting activity of culture media from E. coli cells harboring plasmids encoding wild-type MccA heptapeptide (pA7-ap and pA7) or extended peptides (pA11-ap, pA11, pA15-ap, and pA15). (C) Inhibitory activity of cell extracts obtained from E. coli cells harboring indicated plasmids.

To explain the preference for seven-residue-long MccA precursor peptides in naturally occurring *mcc*-like clusters, two sets of plasmids for the production of E. coli McC or McC-like molecules were created. In the first set, the pA7-ap vector contains the full *mcc* cluster expressed from its natural promoters (Fig. 3A, left) and was expected to produce fully matured McC. The pA11-ap and pA15-ap plasmids are pA7-ap derivatives encoding 11- and 15-amino-acid MccA-based peptides M**GGG**MRTGNAN and MASTA**GGG**MRTGNAN, respectively (the sequence of the natural MccA heptapeptide is underlined). The string of three glycines (highlighted in bold) was chosen to extend the length of the natural heptamer without providing any sequence-specific interactions. To prevent possible artifacts caused by an extensive glycine stretch, a tetrapeptide string (ASTA) composed of small amino acids was incorporated in the A15 peptide. The second set of plasmids (pA7, pA11, and pA15) are pA7-ap and its derivatives lacking the *mccD* and *mccE* genes responsible for installation of the aminopropyl moiety on the peptidyl adenylate (Fig. 3A, right). The E. coli *pepABN* (BW25113 Δ*pepA* Δ*pepB* Δ*pepN*) strain lacking peptidases responsible for intracellular McC processing (2) was used as a production host to avoid intracellular degradation of the peptide moiety of the McC products. Cells transformed with either one of the McC production plasmids grew normally. Antibiotic activity was measured after overnight growth by depositing aliquots of cultured medium on freshly seeded lawns of McC-sensitive E. coli tester cells (Fig. 3B). Clear growth inhibition zones were observed around the points where aliquots of cultured medium from cells carrying pA7-ap, pA7, and pA11-ap were deposited, with growth inhibition zones observed with medium from pA11-ap cells being the smallest. No antibiotic activity was detectable in cultured medium of cells carrying pA11, pA15, or pA15-ap. In what follows, we analyze the reason(s) for the observed dramatic difference in active peptide-nucleotide production caused by the elongation of *mccA*.

### McC variants with longer peptide parts do not accumulate inside producing cells.

We wondered if longer peptides, once modified, are inefficiently exported from the producing cells. In this case, adenylated peptides would accumulate inside cells, since processing of their peptide parts would be prevented by the triple *pepABN* mutation in our production host. Antibiotic activity of extracts prepared from cells carrying *mcc* plasmids encoding MccA of different lengths was determined on McC-sensitive E. coli BL21(DE3) cell lawns (Fig. 3C). Extracts from cells carrying pA7 and pA7-ap plasmids inhibited cell growth, while no growth inhibition activity was detected in extracts from cells carrying plasmids with elongated *mccA* genes. Thus, it appears that either accumulation or bioactivity of elongated peptide adenylates, rather than their export, is compromised.

### The rate of synthesis of *mccA* RNA is not affected by increased length of the *mccA* reading frame.

**FIG 4:**
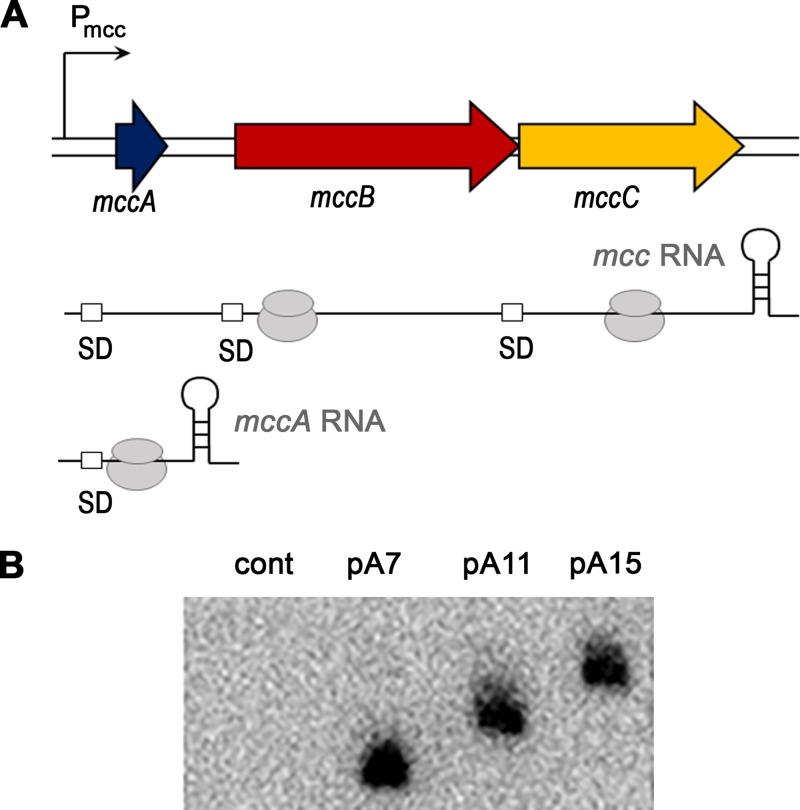
*In vivo* production of *mccA* monocistronic transcripts. (A) The structure of minimized *mcc* operon and expected transcripts. The *mcc* operon promoter P_mcc_ and transcription terminator located between the *mccA* and *mccB* genes are indicated. Ribosomes bound to ORFs are schematically shown. SD, Shine-Dalgarno sequences. Binding of the ribosome to *mccA* induces transcription termination (12). (B) Northern blot hybridization analysis of short monocistronic *mccA* mRNA abundance in cells harboring *mcc* plasmids with different *mccA* gene lengths. Radiolabeled hybridization probe was complementary to an invariant fragment of the *mccA* gene.

We previously showed that efficient synthesis of McC requires transcription termination induced by ribosome binding to the MccA heptapeptide ORF (12) (Fig. 4). This leads to accumulation of ribosome-bound monocistronic *mccA* mRNA from which the MccA peptide is produced in large excess over McC biosynthetic enzymes encoded by downstream genes (12). To establish whether extension of *mccA* disrupts the transcription-translation coupling necessary for *mccA* mRNA production, total RNA was purified from cultures transformed with pA7, pA11, and pA15, and the amount of *mccA* mRNA was determined by Northern blotting. We found similar amounts of *mccA* probe-hybridizing short RNA in each culture (Fig. 4); the transcripts detected in pA11- and pA15-carrying cells were longer than in pA7-carrying cells, as expected. Thus, the observed reduction in the production of microcins with longer peptide parts cannot be explained by lower abundance of longer *mccA* mRNAs.

### Templates encoding longer MccA peptides are translated less efficiently.

**FIG 5:**
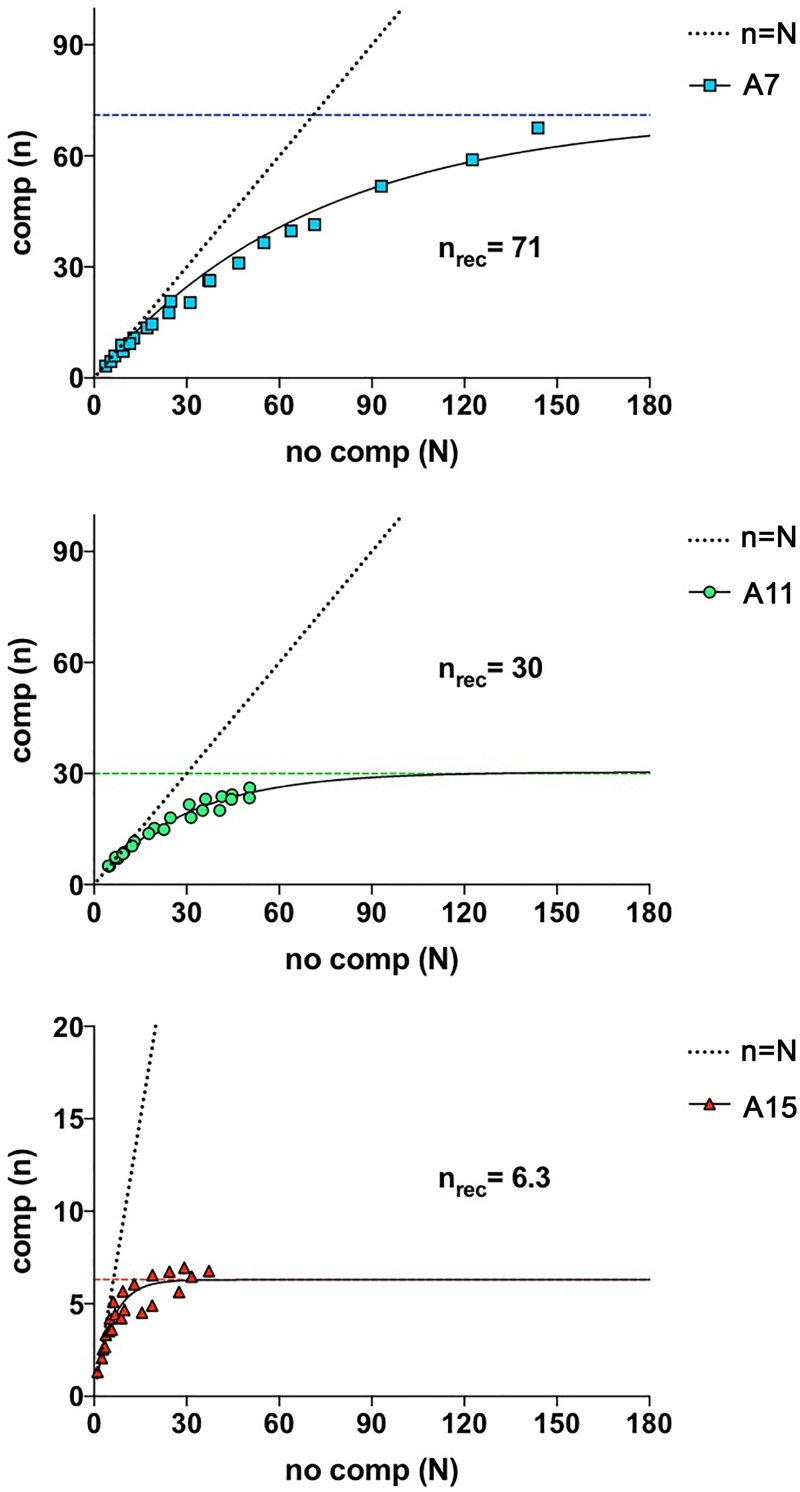
*In vitro* translation efficiency of *mccA* mRNA. Results of translation reiterative initiation experiment with different *mccA* mRNAs are shown. *In vitro* translation reactions in the presence or in the absence of ribosome-sequestering competitor mRNA were performed in parallel. Reaction aliquots were taken at different time points, and the number of MccA peptides synthesized per active ribosome was calculated. Amount of peptide synthesized at each time point without competition (*N*) was plotted versus amount of peptide synthesized with competition (*n*) at the same time point. Dotted lines indicate a case when *n* = *N* (ribosome never leaves mRNA, *n*_rec_ = ∞). Data were fitted according to the model described in reference 13, fitting curves are shown as black lines, and respective *n*_rec_ is shown by the colored dashed lines.

Decreased production of longer MccA peptide adenylates can be caused by lowered synthesis of elongated precursor peptides. It was previously shown that short open reading frames (between 2 and 8 codons) can be reiteratively translated without ribosome dissociation from mRNA (11). Such a mechanism could increase the yield of peptide product per mRNA by bypassing the rate-limiting initiation step in which the *mccA* mRNA is competing with other mRNAs in the cell. The *mccA* gene of E. coli and most other validated McC-like precursor peptide genes are seven codons long and are therefore within the range where reiterative translation can be expected. To check if wild-type *mccA* mRNA can be reiteratively translated by the ribosome without dissociation and exchange with other mRNAs, we used a previously described *in vitro* translation competition assay (11, 13). *In vitro*-synthesized *mccA* mRNA was preincubated with ribosomes, ^3^H-formylmethionyl (fMet)-tRNA^fMet^ (present in excess over ribosomes) (see Materials and Methods), and initiation factors to allow initiation complex formation. Next, components necessary for translation elongation (elongation and termination factors, tRNAs, amino acids and cofactors, and tRNA-recharging system) were added. A 10-fold molar excess (over *mccA*) of competitor mRNA was also added, as needed. The competitor mRNA has a strong ribosome-binding sequence and encodes a peptide whose synthesis is not supported by the mix due to the omission of an encoded amino acid. Thus, once the ribosome is dissociated from the *mccA* template, it is effectively and irreversibly sequestered at the competitor template and no longer able to initiate translation of free *mccA* mRNAs. We monitored the amounts of synthesized MccA peptides per active ribosome as a function of time in the presence (*n*) and in the absence (*N*) of competitor mRNA (13) (see also Materials and Methods for details) and plotted *n* versus *N* in each case, as shown in Fig. 5. If ribosomes do not dissociate from the *mccA* ORF in the course of the experiment, *N* equals *n* (Fig. 5, dotted line). If ribosomes redistribute to competitor template, the MccA peptide synthesis rate decreases with time and *n* becomes less than *N*. The data fitting section below the curves in Fig. 5 allows one to determine *n*_rec_, or ribosome recycle number, i.e., the number of peptide molecules synthesized per active ribosome before ribosome dissociation. An *n*_rec_ value of zero means that the ribosome dissociates after completing the synthesis of the first MccA peptide. As can be seen from Fig. 5, data fitting for the wild-type *mccA* translation (A7) yielded an *n*_rec_ value of 71. Thus, the *mccA* message is translated multiple times before dissociation. The *n*_rec_ values for *mcc* templates encoding longer peptides, A11 and A15, were 30 and 6, respectively. Thus, while extension of the *mccA* ORF does not prevent reiterative translation *in vitro*, it significantly decreases it, which may contribute to the decreased production of longer peptides *in vivo*, although the extent of this effect inside the cells is hard to estimate.

### Formylated heptapeptide MccA is a preferred substrate for MccB.

**FIG 6:**
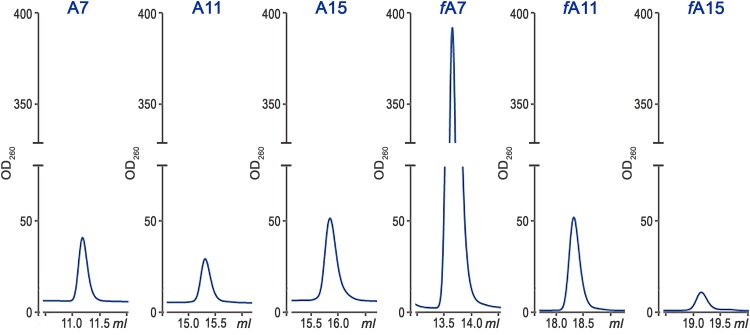
*In vitro* adenylation of MccA peptides of different lengths by MccB. HPLC traces (260 nm) showing product formation after incubation of equal molar amounts of A7 (MRTGNAN), A11 (MGGGMRTGNAN), and A15 (MASTAGGGMRTGNAN) peptides and their N-terminally formylated variant peptides (marked with the *f* prefix) with MccB in the reaction buffer for 20 min at 28°C.

In previous work, it was shown that E. coli MccB is able to modify a wide range of substrates *in vivo* and *in vitro*, including MBP protein with attached A7 peptide (14). To test MccB activity on elongated substrates encoded by pA11 and pA15, we incubated purified MccB with ATP and chemically synthesized A11 and A15 peptides and measured adenylated peptide yields by high-pressure liquid chromatography (HPLC) (Fig. 6). As can be seen, comparable yields of the adenylated MccA A7, A11, and A15 variants were observed. We next repeated the experiment with N-terminally formylated versions of each peptide that correspond to the products of ribosomal synthesis. Formylation of the A7, A11, and A15 peptides resulted in an approximately 8-fold increase, 2-fold increase, and 3-fold decrease in adenylated product yields compared to the yields obtained with A7, A11, and A15, respectively. We conclude that under our conditions, the presence of the formyl group on the N-terminal methionine residue selectively amplified the yields of MccA heptapeptide adenylation compared to adenylation yields of longer peptides A11 and A15.

### Bioactivity of McC-like compounds is affected by the length of the peptide chain and the presence of the N-terminal formyl group.

**FIG 7:**
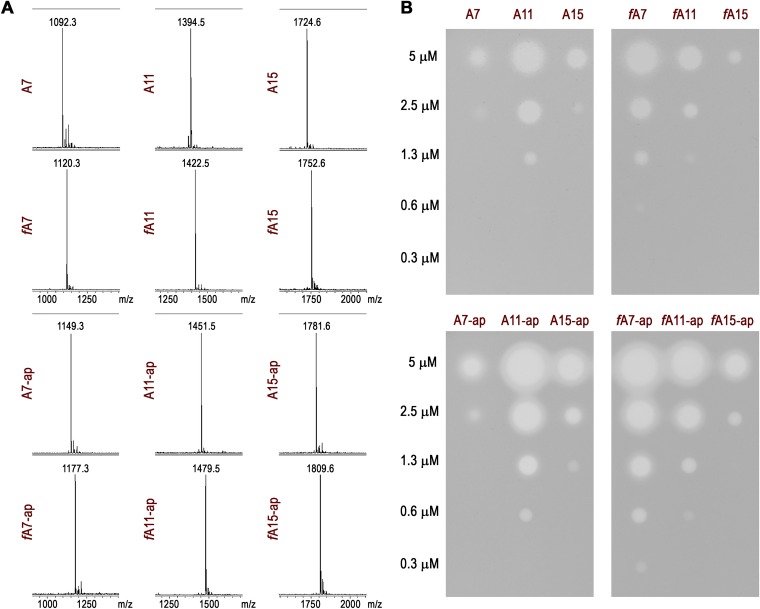
Bioactivity of elongated McC-like compounds. (A) MALDI-TOF MS spectra of *in vitro-*synthesized microcin species. A7 (MRTGNAN), A11 (MGGGMRTGNAN), and A15 (MASTAGGGMRTGNAN) peptides and their N-terminally formylated variants (marked with the *f* prefix) were adenylated and aminopropylated *in vitro*. ap, aminopropyl. (B) MICs of indicated microcin species measured by the spot test on the lawns of McC-sensitive E. coli BL21(DE3) cells.

We used recombinant E. coli MccB adenylate transferase and the cognate MccD/MccE enzymes pair to produce aminopropylated McC and its longer peptide derivatives *in vitro*. Chemically synthesized formylated and desformylated MccA-based peptides were used as the substrates. Reaction products were purified as a single HPLC peak, and their identity was confirmed by matrix-assisted laser desorption ionization–time of flight mass spectrometry (MALDI-TOF MS) (Fig. 7A). When 10 μM solutions of each compound were tested for antibiotic activity, clear growth inhibition zones were observed in all cases (Fig. 7B). MIC measurements performed using serial dilutions of each compound revealed that the desformylated adenylate of the 11-amino-acid peptide was 2-fold more active than the corresponding adenylated heptapeptide A7-McC, while activity of the adenylated A15 peptide was similar to that of A7-McC (Fig. 7B, top left panel). Aminopropylation increased the toxicity of each of the peptidyl adenylates by ca. 2-fold (Fig. 7B, top versus bottom panels). The formylation of the N terminus had a more complex effect on bioactivity. It slightly reduced the MICs of both elongated peptide adenylates (with or without aminopropyl) but stimulated, by ca. 4-fold, the activity of heptapeptide adenylates (Fig. 7B, left versus right panels). Overall, it appears that while either formylation of the N terminus or limited elongation of the 7-amino-acid MccA peptide leads to increased bioactivity of the resulting peptide adenylate, there is no synergy between these two effects and formylation makes a larger contribution to bioactivity than peptide length.

## DISCUSSION

The primary impetus for this work came from our desire to understand why many *mcc* operons from diverse bacteria encode peptide precursors that are 7 amino acids in length (Fig. 2). A phylogenetic analysis revealed a well-supported large clade of MccB family proteins associated with either validated or predicted precursor peptides. Most precursors were 7 amino acids long, suggesting that peptide length is a conserved and potentially functionally important feature of microcin C-like compounds. Previously, we showed that in the case of E. coli McC, a decrease of the peptide moiety length by just 1 amino acid prevented facilitated transport by the YejABEF transporter into sensitive cells, thus abolishing activity (15). Conversely, increasing the peptide moiety length appeared to have a marginal effect on the bioactivity of McC derivatives prepared by enzymatic synthesis *in vitro* (10), indicating that there may not be a stringent upper limit of precursor peptide length. Surprisingly, we here observed that increasing the length of the E. coli *mccA* peptide precursor gene has a dramatic negative effect on the antibiotic activity of producing cells. Given that McC biosynthesis and the mechanism of action are well understood, this activity decrease could *a priori* have been due to (i) lower rates of synthesis of longer MccA peptides, (ii) less efficient adenylation of longer peptides, (iii) impaired export of mature compounds, (iv) decreased antibacterial activity of products with longer peptides, or any combination of these factors. We experimentally tested each one of the possibilities listed above and found that the length of the MccA product contributes to McC production and activity on multiple levels. Somewhat surprisingly, increasing the length of the *mccA* open reading frame had no effect on the transcription-translation coupling in the *mcc* operon. This follows from the observation that the termination mechanism that ensures increased accumulation of *mccA* transcript over the transcripts of McC biosynthesis genes is not affected by increasing the *mccA* gene length from 7 to 15 codons. At the same time, translation of *mccA* mRNA does indeed depend on the length of the ORF. Specifically, we showed that mRNA encoding a 7-amino-acid MccA peptide very efficiently underwent reiterative translation in the presence of competitor RNA. This ability to withstand the competition and reinitiate translation without ribosome dissociation was decreased (but not abolished) by increasing the *mccA* reading frame up to 15 codons. While at present it is not possible to assess the significance of *in vitro*-determined numbers of synthesis rounds without dissociation for *in vivo* MccA synthesis, it is reasonable to assume that such reiterative translation contributes to higher yields of heptameric MccA and, hence, to the increased yield of the final product.

Unexpectedly, the largest contributor to preferential synthesis of wild-type McC *in vitro*, compared to that of elongated peptide derivatives, is the formyl group present at the N-terminal methionine. This group leads to an almost 10-fold increase in the production yield of adenylated heptapeptide but has a much smaller or even inhibitory effect on the adenylation of 11- and 15-amino-acid-long derivatives. The observed strong stimulation of adenylation by a formyl group on the heptapeptide MccA is fully in line with recent structural results that show the existence of a special binding pocket for the N-terminal formyl in the MccB enzyme (16). The presence of a formyl group in longer peptides is not expected to have a stimulatory effect, since the positioning of the C-terminal aspartate in the MccB catalytic site allows only the N-terminal backbone residue positioned 7 amino acids away to make favorable interactions with the enzyme.

The MccD/E enzymes required for the secondary modification of peptide nucleotide phosphate with aminopropyl are encoded by both long and short MccA *mcc* operons (17). It is therefore an expected result that this secondary modification had no specific effect on bioactivity of McC-like compounds with different peptide lengths and generally increased the level of bioactivity. In contrast, formylation acts as a specific contributor to the activity of adenylated heptameric peptides. The presence of the formyl group makes natural E. coli McC a better antibacterial agent than either 11- or 15-amino-acid peptide adenylate with or without aminopropyl. We speculate that this effect may be due to better transport of formylated peptides of appropriate length by the YejABEF transporter, which was previously shown to recognize formylated peptides (15). In the absence of the N-terminal formyl group, increasing the peptide length up to a certain point also increases the bioactivity (14). Interestingly, in the case of the Yersinia pseudotuberculosis *mcc* operon, which encodes a 42-amino-acid-long MccA precursor, the final peptide-nucleotide product is processed to an 11-amino-acid peptide with attached C-terminal nucleotide, and this processing is required for bioactivity (17). We speculate that such processing may be a common feature of *mcc* operons with longer MccA precursors.

## MATERIALS AND METHODS

### Phylogenetic analysis.

The initial data set consisted of 12 MccB homologs forming a branch on MccB/PaaA family tree that contained validated microcin C-like clusters (10). Protein sequences were retrieved from the NCBI protein database (https://www.ncbi.nlm.nih.gov/protein/) and aligned using MUSCLE v3.8.31 with defaults (18). Since the N-terminal RiPP precursor peptide recognition element (RRE) domain of MccB proved to be critical for microcin C processing (19), it was employed to distinguish the MccB subfamily from other members of the ThiF superfamily (20). The N-terminal 86-amino-acid portion of the alignment containing the RRE domain was used as a query for a PSI-BLAST search against protein reference sequences downloaded on 22 December 2018 (21). PSI-BLAST was iterated until convergence with an inclusion E value threshold of 0.005 and maximum of 500 hits. To remove redundancy, the resulting 202 MccB homologs were clustered using MMseqs2 with identity and coverage cutoffs of 0.95 and 0.9, respectively (22). One random representative sequence for each of 82 clusters was chosen for further analysis. Genomic regions of MccB homologs were analyzed with RODEO to check the presence of ThiF domain and a transport-related gene within corresponding gene cluster (23).The final set of MccB homologs was aligned with MUSCLE. Partial sequences were removed. The alignment was trimmed and extremely variable columns were removed. A maximum likelihood phylogenetic tree of the 71 remaining sequences was estimated using RAxML with an LG amino acid substitution model and gamma-distributed evolutionary rates (24). The LG substitution matrix was chosen for phylogenetic analysis according to the Akaike information criterion implemented in ProtTest 3.0 (25). Rapid bootstrap analysis and a search for the best tree were performed in a single run. Bootstrapping converged after 400 replications according to the autoMRE criterion. The tree was rooted using an outgroup consisting of PaaA proteins that have the same domain architecture but different function (20). The tree was then visualized using iTOL (26). The precursor peptides for each putative microcin C cluster were manually extracted from the corresponding genomic records on the basis of the following criteria: ORF with a strong ribosome binding site and C-terminal asparagine in the close proximity (as far as 300 bp from gene cluster) of MccB preferentially upstream of MccB and on the same strand.

### Bacterial strains and vectors.

E. coli strain BW25113 *ΔpepA ΔpepB ΔpepN* was used as McC producer. An E. coli BL21(DE3) strain was used as the McC-sensitive strain for growth inhibition tests. To create pA11-ap and pA15-ap, encoding extended *mccA*, double-stranded DNA (dsDNA) containing the extended *mccA* gene was prepared by two oligonucleotides annealing and inserted between SalI and NsiI of the pA7-ap vector (pp70 plasmid [12]). The pA7, pA11, and pA15 vectors were constructed from the corresponding pA7-ap, pA11-ap, and pA15-ap plasmids by the deletion of *mccD*, *mccE*, and *mccF* genes using Gibson Assembly Mix (NEB, USA).

### Bioactivity assays.

McC activity assay was performed as described previously (14), with a freshly seeded lawn of sensitive cells prepared from E. coli BL21(DE3) cells on M9 medium.

To determine intracellular McC content, the cell pellet obtained from 100 ml of overnight cell culture was resuspended in 5 ml of 1× phosphate-buffered saline and disrupted by ultrasonication. The lysate was cleared by centrifugation, mixed with an equal volume of 0.1% trifluoroacetic acid (TFA) in acetonitrile, and incubated on ice for 30 min. The insoluble fraction was removed by centrifugation. The supernatant was vacuum dried, and the resulting pellet was dissolved in 100 μl of water and cleared by centrifugation. Bioactivity was checked by applying 10-μl drops of the cleared supernatant on the freshly prepared lawn of sensitive cells.

### *In vitro* synthesis of McС*-*like compounds.

Synthetic peptides were purchased from GenScript Biotech Corp., USA. E. coli MccB was purified as described previously (10). Briefly, the BL21(DE3) cells transformed with pET-MccB vector (10) were grown at 37°C in 200 ml of LB medium supplemented with 50 μg/ml ampicillin until the optical density at 600 nm reached 0.6. Protein production was induced with 0.1 mM isopropyl β-d-1-thiogalactopyranoside (IPTG) followed by growth at 18°C for 16 h. Cells were harvested and resuspended in 8 ml of BW buffer (20 mM Tris-HCl [pH 8.0], 500 mM NaCl, and 1 mM β-mercaptoethanol) supplemented with 0.2 mM phenylmethanesulfonyl fluoride (PMSF) and 1 mg/ml lysozyme. Cells were disrupted by sonication, and the insoluble fraction was precipitated by centrifugation at 30,000 × *g* for 30 min at 4°C. The clarified cell lysate was loaded onto a 1-ml HiTrap Co^2+^-chelating HP Sepharose column (GE Healthcare) preequilibrated with BW buffer. The column was washed with 10 ml of BW buffer, and the MccB protein was eluted with EB buffer (50 mM Tris-HCl [pH 8.0], 0.5 M imidazole, 300 mM NaCl, 10% glycerol). Protein fractions were analyzed using Laemmli 10% SDS-PAGE. Protein concentration was measured according to the Bradford method.

For elongated McC-like compound synthesis, 100 μM peptides were adenylated by 12 μM MccB in the mixture with 100 mM Tris (pH 7.5), 50 mM NaCl, 10 mM MgCl_2_, 5 mM ATP, and 10 mM dithiothreitol (DTT) at 32°C for 8 h. Reaction mixtures were checked by MALDI-TOF MS for reaction completion, and compounds were purified by HPLC using an Eclipse Plus C_18_ column (4.6 mm by 250 mm; particle size, 5 μm; Agilent Technologies) and 0% to 15% linear elution gradient of acetonitrile in 0.1% TFA. The fractions were vacuum dried and dissolved in deionized water for concentration measurements. The published extinction coefficient for AMP (E^mM^ = 15.4 at 259 nm in 0.01 M PO_4_ [pH 7.0]) was used to calculate peptidyl nucleotide concentrations (27).

For adenylation efficiency measurements, 500 μM peptide was incubated with 5 μM MccB at 28°C for 20 min, and then the reaction was quenched with 0,1% TFA and analyzed using C_18_ reversed-phase chromatography (Symmetry C_18_, 5 μm, 4.6 mm by 150 mm; Waters). A 0% to 15% linear gradient of acetonitrile in 0.1% TFA was applied for elution. The identities of the compounds in the chromatographic fractions were confirmed by MALDI-TOF MS.

Purification of MccD and MccE enzymes and *in vitro* aminopropylation of the compounds were performed as described in reference 6.

### MALDI-TOF MS analysis.

Sample aliquots (1 to 2 μl) were mixed on a steel target with 0.5 μl of 20 mg/ml 2,5-dihydroxybenzoic acid in 0.5% TFA and 30% acetonitrile (ACN) water solution (Aldrich). Mass spectra were recorded on an UltrafleXtreme MALDI-TOF/TOF mass spectrometer (Bruker Daltonics) equipped with a neodymium laser. The [MH]^+^ molecular ions were measured in reflector mode; the accuracy of mass peak measurement was within 0.1 Da.

### The *mccA* transcript detection.

An aliquot of an overnight culture was diluted 100-fold with LB containing 100 μg/ml carbenicillin and grown for 4 h at 37°C (OD_600_ reached approximately 2.0). Cells from 0.5- to 3-ml culture aliquots were collected by centrifugation, the supernatant was discarded, and cell pellets were frozen in liquid nitrogen and stored at −70°C until further use. Cell pellets were resuspended in 30 to 50 μl of 20 mM Tris-HCl (pH 8.0) with 1 mM EDTA, treated with 10 mg/ml lysozyme, mixed with 500 μl of TRIzol reagent (Invitrogen), and incubated for 5 min at room temperature. One hundred microliters of chloroform was added, and the samples were incubated for 15 min on ice until phase separation occurred. After a 15-min centrifugation at 13,000 × *g*, the aqueous phase (∼300 μl) was transferred to a fresh tube. RNA was ethanol precipitated and then redissolved in 20 to 50 μl of diethyl pyrocarbonate (DEPC)-treated water. RNA concentration was determined using a NanoDrop 2000 spectrophotometer (Thermo Scientific, USA).

For Northern blot hybridization, the RNA samples were diluted to a final concentration of 0.25 μg/μl with formamide loading buffer (95% formamide, 0.025% xylene cyanol, 0.025% bromophenol blue), and 1 μg of RNA was loaded on an 8% (19:1) denaturing polyacrylamide gel. After electrophoretic separation, RNA was electroblotted to a Hybond XL membrane (Amersham) using a wet transfer system (Bio-Rad). The transfer was performed for 1 h at 250 mA in 0.5× Tris-borate-EDTA (TBE) buffer. The membrane was air dried and baked using “optimal cross-link” settings of the UV cross-linker (UVP). The membrane was blocked with 3 ml of ExpressHyb buffer (Clontech) for 30 min at 37°C; 20 pmol of 5′ ^32^P-labeled oligonucleotide probe was added, and hybridization was performed overnight at 37°C. The membrane was washed twice for 10 min with 2× SSC (1× SSC is 0.15 M NaCl plus 0.015 M sodium citrate) with 0.1% SDS and once for 10 min with 0.1× SSC with 0.1% SDS. The results were visualized using Storm Phosphorimager (GE Healthcare).

### *In vitro* translation.

DNA templates were prepared by PCR with a universal forward primer containing the T7 promoter sequence. RNA templates were prepared by transcription with T7 polymerase (40 mM Tris [pH 8], 20 mM MgCl_2_, 5 mM spermidine, 25 mM DTT, 0.01% Triton X-100, 5 mM ATP, 4 mM each GTP, UTP, and CTP, 0.02 μM T7 polymerase, 0.005 U/μl inorganic pyrophosphatase, 1 U/μl inhibitor of RNases, and 1 ng/μl DNA). After RNA synthesis, the reaction mixture was treated with DNase I (1 U per 250 μl reaction mixture) for 15 min, phenol extracted twice, and applied to a NAP 10-gel filtration column (GE Healthcare) to remove unincorporated nucleotides. The eluate was precipitated, redissolved in water, and used as a template for *in vitro* translation.

The DNA templates used (T7 promoter shown in lowercase, RNA parts in uppercase, ribosome binding site [RBS] positions underlined, ORF positions in boldface font, and stem-loop positions in underlined italics) are as follows: A7 (peptide MRTGNAN), ttaatacgactcactatagGTGAATTAATCACAGTAATAGGAGGGTCGAC**ATGCGTACTGGTAATGCAAACTAA**TGGCAAAATATAAATGTCCATTAA*ATGCCACCCT*GTAC*AGGGTGGCAT*ACAAATGCATTCTGCGAGGTTTATTTATGGATTATATATTGGGTCGCTATGTC; A11 (peptide MGGGMRTGNAN), ttaatacgactcactatagGTGAATTAATCACAGTAATAGGAGGGTCGAC**ATGGGTGGCGGTATGCGTACTGGTAATGCAAACTA**ATGGCAAAATATAAATGTCCATTAA*ATGCCACCCT*GTAC*AGGGTGGCAT*ACAAATGCATTCTGCGAGGTTTATTTATGGATTATATATTGGGTCGCTATGTC; A15 (peptide MASTAMGGMRTGNAN), ttaatacgactcactatagGTGAATTAATCACAGTAATAGGAGGGTCGAC**ATGGCGAGCACCGCGGGTGGCGGTATGCGTACTGGTAATGCAAACTAA**TGGCAAAATATAAATGTCCATTAA*ATGCCACCCT*GTAC*AGGGTGGCAT*ACAAATGCATTCTGCGAGGTTTATTTATGGATTATATATTGGGTCGCTATGTC; and 02.MFF.Oa competitor template (peptide MFF), ttaatacgactcactatagGGGAATTCGGGCCCTTGTTAACAATTAAGGAAGGTATACT**ATGTTTTTCTAA**CTGCAGAAAAAAAAAAAAAAAAAAAAA.

An optimized *in vitro* translation system described in reference 28 was used in competition mode (11). Briefly, two mixtures were prepared in HEPES-polymix buffer (30 mM HEPES, 5 mM magnesium acetate [MgOAc], 5 mM NH_4_Cl, 95 mM KCl, 0.5 mM CaCl_2_, 8 mM putrescine, 1 mM spermidine, and 1 mM DTT) with additional 1 mM MgOAc, 1 mM ATP, 10 mM phosphoenolpyruvate (PEP), and 1 mM GTP. The ribosome mixture contained ribosomes (0.05 μM or more active ribosomes), [^3^H]-fMet (30- to 90-fold molar excess over the active ribosomes, depending on the number of cycles required), 1 μM protein initiation factor 1 (IF1), 0.5 μM IF2, 0.5 μM IF3, and *mcc* mRNA (2- to 5-fold excess over the active ribosomes). The factor mixture contained 200 μM each amino acid used with the exception of Ala (600 μM), 2 U/μl aminoacyl-tRNA synthetases, 40 μM bulk E. coli tRNA, 2 μM EF-G, 20 μM EF-Tu, 2 μM EF-Ts, 1 μM each replicative form 2 (RF2) and RF3, 6 μM RRF, 50 ng/μl pyruvate kinase, and 3 ng/μl myokinase. All components of the mixture were purified as described in reference 27. In reactions with competition, a 10-fold excess of the competitor template over *mcc* mRNA was added. The 02.MFF.Oa template encoding MFF tripeptide (11) was used as a competitor. No Phe was added in the reaction mix.

Both mixtures (60 to 120 μl) were preincubated at 37°C for 10 min and mixed together to start translation elongation at 37°C. To monitor the progress of the translation reaction, 13-μl aliquots were taken at different time points after the reaction start and quenched with one-third volume of 50% formic acid (to make final acid concentration 17%). The quenched samples were incubated on ice for 5 min to precipitate the components of the translation reaction and then centrifuged for 15 min at 4°C. The supernatants, containing the released peptides, were separated by HPLC on a C_18_ reversed-phase column (LiChrospher 100 RP-18 [5 μm], LiChroCART 125-4; Millipore). Samples were separated with a linear gradient from 10% to 70% methanol (MeOH) with 0.1% TFA for 40 min at 0.45 ml/min flow. Radioactivity was monitored by an online radiometer (Ramona), and the amounts of synthesized peptides were quantified by peak integration.

### Translation data fitting.

In the absence of mRNA competition the per ribosome amount [*y*(*t*)] of synthesized peptide at incubation time *t* is given by y(t)=1+k⋅t=1+N. In the presence of mRNA competition the corresponding amount [*y_c_*(*t*)] of synthesized peptide is given by Dincbas et al. (11): $y_{c}(t)=1+\left((1-P)/P\right)\cdot (1-e^{-P\cdot k\cdot t})=1+n$. Here, *P* is the probability that mRNA is released from the ribosome at any single recycling step. Note that here *y* and *y_c_* describe experimental situations in which a first peptide is rapidly formed with a probability of 1, while subsequent peptide chains require absence of mRNA release in proceeding recycling reactions. From this, it follows that (see Fig. 5) $n=\left((1-P)/P\right)\cdot (1-e^{-P\cdot N})$. It follows that the average number of peptide formation cycles before a given mRNA dissociates from the ribosome, *n*_rec_, is given by $n_{\text{rec}}=(1-P)/P$. For small values of *N* and *P*, respectively, it follows from Taylor expansions that n≈{N≪1}≈(1−P)⋅N≈{P≪1}≈N.
